# Stress Affects Mast Cell Proteases in Murine Skin in a Model of Atopic Dermatitis-like Allergic Inflammation

**DOI:** 10.3390/ijms25115738

**Published:** 2024-05-24

**Authors:** Frank R. Rommel, Susanne Tumala, Anna-Lena Urban, Frank Siebenhaar, Johannes Kruse, Uwe Gieler, Eva M. J. Peters

**Affiliations:** 1 Psychoneuroimmunology Laboratory, Department of Psychosomatic Medicine and Psychotherapy, Justus Liebig University Giessen, 35390 Giessen, Germany; 2 Institute of Allergology, Charité-Universitätsmedizin Berlin, Corporate Member of Freie Universität Berlin and Humboldt-Universität zu Berlin, 12203 Berlin, Germany; 3 Fraunhofer Institute for Translational Medicine and Pharmacology ITMP, Immunology and Allergology, 12203 Berlin, Germany; 4 Department of Psychosomatic Medicine and Psychotherapy, Justus Liebig University Giessen, 35390 Giessen, Germany; 5 Department of Dermatology, University Hospital Giessen, 35392 Giessen, Germany; 6 Charité Center 12 for Internal Medicine and Dermatology, Charité-Universitätsmedizin Berlin, 10117 Berlin, Germany

**Keywords:** mouse mast cell protease (mMCP), stress, atopic dermatitis-like allergic inflammation (AlD), non-neuronal cholinergic system (NNCS), nicotinic acetylcholine receptor α7 (Chrna7), secreted Ly-6/uPAR-related protein 1 (SLURP1), stress, substance P (SP)

## Abstract

Stress exposure worsens allergic inflammatory diseases substantially. Mast cells (MCs) play a key role in peripheral immune responses to neuroendocrine stress mediators such as nerve growth factor (NGF) and substance P (SP). Mast cell proteases (MCPs) and cholinergic factors (Chrna7, SLURP1) were recently described to modulate MC stress response. We studied MCPs and Chrna7/SLURP1 and their interplay in a mouse model for noise induced stress (NiS) and atopic dermatitis-like allergic inflammation (AlD) and in cultured MC lacking Chrna7. We found that the cholinergic stress axis interacts with neuroendocrine stress mediators and stress-mediator cleaving enzymes in AlD. SP-cleaving mMCP4+ MC were upregulated in AlD and further upregulated by stress in NiS+AlD. Anti-NGF neutralizing antibody treatment blocked the stress-induced upregulation in vivo, and mMCP4+ MCs correlated with measures of AlD disease activity. Finally, high mMCP4 production in response to SP depended on Chrna7/SLURP1 in cultured MCs. In conclusion, mMCP4 and its upstream regulation by Chrna7/SLURP1 are interesting novel targets for the treatment of allergic inflammation and its aggravation by stress.

## 1. Introduction

Stress triggers the release of a wide variety of neuroendocrine mediators and thereby influences immune activation [1]. Neuro-immune communication leads to neurogenic inflammation and its augmentation by stress in many diseases characterized by maladaptive immune activation, such as allergic and autoimmune disorders. Mast cells (MCs) are at the center of this interaction. They are strategically placed in organs at the border between the body and its environment [2,3,4]. In organs rich in MC, such as the skin, they are found near nerve fibers, and the neuroendocrine mediators that nerve fibers release enhance MC activity. This close connection links MC-rich peripheral organs with the brain and is known as the brain–skin axis [2,4,5,6]. Most notably, MCs are responsible for quick innate host defense [7,8], but in chronic inflammatory diseases, MC hyperactivation plays a deleterious role by triggering flares and worsening disease trajectories.

Sensory neuron-derived neuropeptides are established activators of MCs in a wide variety of triggering situations, ranging from biomolecular to psychosocial stress. Substance P (SP) is perhaps the best studied neuropeptide in this context [7,9]. The neuropeptide calcitonin gene-related peptide (CGRP) is frequently co-expressed with SP [10]. CGRP is a potent vasodilator and plays a role in the recruitment of inflammatory cells to sites of inflammation. Together, they induce and maintain neurogenic inflammation. Upstream of neuropeptides, the stress-inducible neurotrophin nerve growth factor (NGF) and brain-derived neurotrophic factor (BDNF) closely link the local neurogenic stress response to the brain and establish the circuitries of the brain–skin axis [11,12].

When an acute immune challenge has successfully been battled, neurogenic inflammation must be terminated to prevent inflammatory tissue damage. If MC activation is not terminated, neurogenic inflammation continues, which contributes to the development of inflammatory disease and its exacerbation. Respective neuropeptide-driven pathomechanisms are well studied in atopic dermatitis. Related animal models were shown to be highly sensitive to stress-induced exacerbation [13,14]. In a mouse model for atopic dermatitis-like allergic inflammation (AlD) established in our laboratory, 24 h of noise-induced stress (NiS) led to the release of SP, which triggered MC degranulation with deleterious pro-inflammatory effects and a doubling of skin inflammation [15] in an neurokinin I (NK1)- and NGF-dependent manner [16]. This initial finding has since been confirmed by a number of studies [16,17,18,19].

Interestingly, MC store and release proteins, called mast cell proteases (MCPs). MCPs cleave and thereby terminate the activity of many stress mediators. MCPs regulate the intensity and duration of neuropeptide-induced inflammatory responses and may thereby contribute to their timely termination [20,21,22]. In the context of neurogenic inflammation, mouse MCP4 (mMCP4) (highest homology to human chymase) cleaves SP, whereas mMCP6 (highest homology to human tryptase) cleaves CGRP [23]. This suggests that MC may have the ability not only to initiate but also to stop neurogenic inflammation. If this stop signal is missing, inflammation may continue unhindered, contributing to the often-described vicious cycle between stress and disease aggravation so severely diminishing the quality of life in affected patients [24,25].

Recently, so-called anti-inflammatory neuroendocrine-immune circuits have gained considerable attention, and it has become evident that allergic inflammatory diseases are characterized by altered cholinergic signaling [26,27,28,29]. The nicotinergic acetylcholine receptor alpha 7 (Chrna7) downregulates tumor necrosis factor alpha (TNFα) production by macrophages [30] and was also found on MC [31,32,33]. Activation of Chrna7 was shown to downregulate MC inflammatory responses and hence to establish resilience against hyperinflammatory responses [32]. Surrounding cells and nerve fibers produce and release Chrna7 ligands such as acetylcholine, which aggravates T helper cell type 2 (TH2)-driven inflammation, itch, and MC degranulation [28]. However, they also produce and release the neuropeptide Ly-6/uPAR-related protein 1 (SLURP1), an allosteric endogenous Chrna7 ligand of Chrna7 able to modify its activation [34]. Under conditions of stress, SLURP1 upregulates innate and T helper cell type 1 (TH1) immunity in skin [29]. Whether the interplay between SLURP1 and Chrna7 plays a role in the stress cascade along the brain–skin axis in allergic inflammation and whether this involves regulation of stress mediator availability by regulation of MCP have not been studied to date.

This knowledge gap prompted us to investigate whether stress deregulates MCP expression in MC and whether this involves Chrna7 and its endogenous ligand SLURP1. To follow up on this hypothesis, we first analyzed the expression and localization of mMCP proteins in the skin of stressed AlD mice and compared the expression to unstressed, stressed, and AlD mice as well as mice treated with a stress-effect neutralizing antibody to the nerve growth factor (NGF) [16]. In addition, cultured MCs were used to analyze the interaction between SP and SLURP1 with respect to MC activation and mMCP production.

## 2. Results

### 2.1. Immunohistomorphometry Reveals Stress-Induced Increase of mMCP4+ MCs in AlD

**Figure 1:**
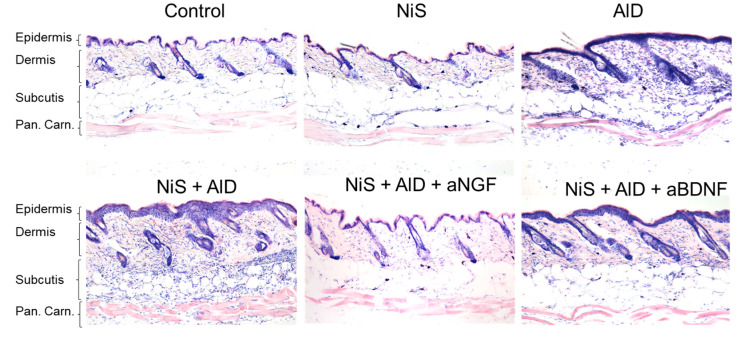
Stress (NiS) and allergic inflammation (AlD) combined dramatically enhance skin inflammation in murine back skin. Giemsa staining shows exemplary back skin full-thickness biopsies. Sections were performed along the head–tail axis as evidenced by the telogen hair follicles visible. AlD skin shows thickening of the epidermis and an increased cell density in dermis and subcutis in evidence of inflammatory infiltration. In the back skin of mice that were additionally stressed, both epidermal thickness and infiltration are dramatically increased, which is not the case in the back skin samples additionally treated with neutralizing antibodies against NGF (aNGF) or BDNF (aBDNF). Abbr.: AlD = atopic dermatitis-like allergic inflammation; BDNF = brain-derived neurotrophic factor; NGF = nerve growth factor; NiS = noise-induced stress; Pan. Carn.: = panniculus carnosus.

Employing our established AlD model by provoking an allergic dermatitis-like inflammation in murine skin [1,15,16,17,29,35,36], we confirmed the previously described worsening of skin inflammation by stress and its reduction in the presence of NGF-neutralizing antibodies. We here also show a mild reduction by treatment with BDNF-neutralizing antibodies. In this model, allergic inflammation is provoked by intradermal injection of the allergen rather than transcutaneous application to allow precise timing of the stress hit and the inflammation hit. The resulting epidermal thickening and dermal/subcutaneous infiltration can be observed by Giemsa staining of full-thickness skin biopsies (Figure 1).

**Figure 2:**
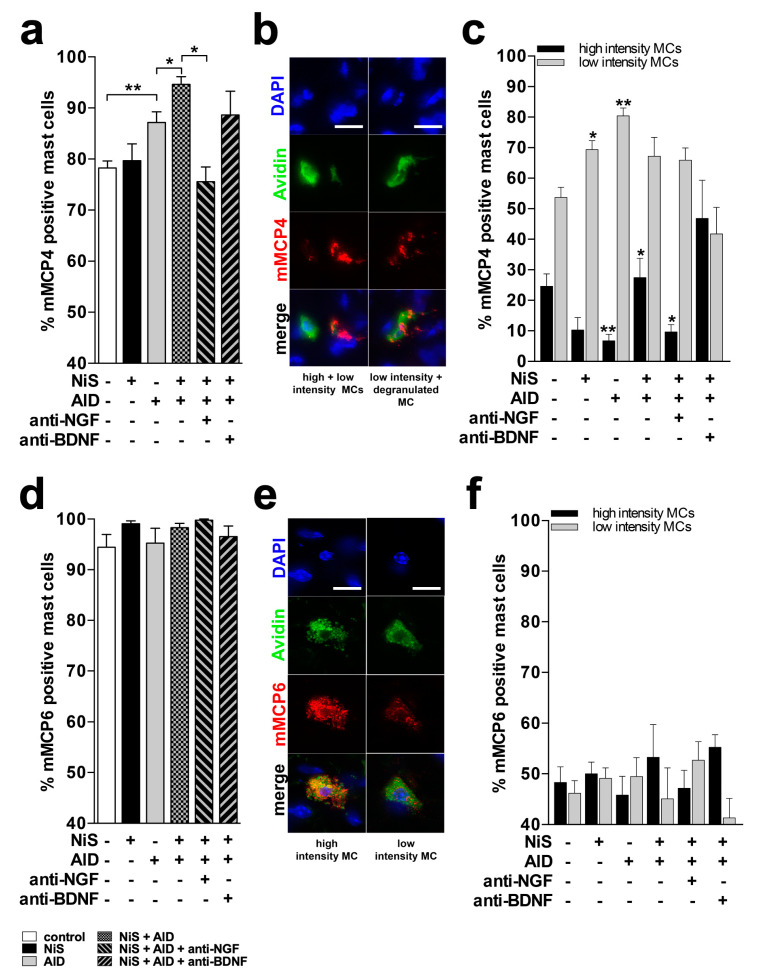
Stress and stress mediator effects on mMCP protein and mRNA expression in MCs. Data represent the mean values of the results from 5 animals (*n* = 5). ** = *p* ≤ 0.01; * = *p* ≤ 0.05. Immunohistomorphometric analysis was performed in 10 microscopic fields per animal on 14 µm thick skin cryosections for (**a**,**d**) mMCP4+ and mMCP6+ MCs (expressed in % of total number of FITC-Avidin+ MCs). FITC-Avidin is used as a specific MC marker in skin. (**c**,**f**) High- (black bar) and low-intensity (gray bar) staining of mMCP4+ and mMCP6+ MCs (sums up to % of mMCP+ MCs of the respective group given in (**a**,**d**)). (**b**,**e**) Representative photomicrographs of an immunohistochemical triple staining for mMCP4 and mMCP6 (red), MCs (FITC-labeled avidin, green), and cell nuclei (DAPI, blue), scale bar = 10 µm. Abbr.: AlD = atopic dermatitis-like allergic inflammation; anti-BDNF = antibody against BDNF; anti-NGF = antibody against NGF; BDNF = brain-derived neurotrophic factor; MC = mast cell; mMCP = murine mast cell protease; NGF = nerve growth factor; NiS = noise induced stress.

Immunofluorescence of full thickness back skin biopsies confirmed mMCP4 and mMCP6 protein expression in cutaneous MC (Figure 2b). Quantitative histomorphometry revealed that there was no difference in the percentage of mMCP4-positive (mMCP4+) MC between NiS and controls (Figure 2a). However, induction of AlD resulted in a higher percentage of mMCP4+ MC compared to controls (Figure 2a). NiS in combination with AlD further increased the percentage of mMCP4+ MCs (Figure 2a).

### 2.2. Immunohistomorphometry Suggests mMCP4 Production and Release

The intensity of mMCP expression may be related to differences in the production and release of mMCPs. Therefore, we performed a detailed analysis of mMCP4 and mMCP6 expression intensities within the mMCP+ population. All mMCP+ MCs were classified as high if >50% and low if <50% of the area of a cell was immunoreactive (Figure 2b). This analysis revealed that NiS decreased the percentage of high- and increased the percentage of low-intensity MCs compared to control, indicating increased release of mMCP4. In AlD compared to the control, a similar decreased percentage of high- and increased percentage of low-intensity MCs was detected. NiS+AlD resulted in an increase of high-intensity MCs compared to AlD alone (Figure 2c).

### 2.3. Blocking NGF Reduces mMCP4+ MC in NiS+AlD Skin

To test the functional relevance of mMCP4 upregulation we next used NGF and BDNF neutralizing antibodies, as this treatment has been shown to successfully neutralize neurogenic inflammation effects in allergic dermatitis [16]. Blocking NGF in NiS+AlD mice with i.p. injected neutralizing antibodies normalized the percentage of mMCP4+ MC, while additional treatment with aBDNF in NiS+AlD had no effect on mMCP4 expression in MC (Figure 2a). Similarly, the effects of NiS on high and low percentages of mMCP4+ MC in AlD MCs were reversed by NGF blockade. Blocking of BDNF revealed no significant difference in mMCP4 expression in MC (Figure 2b,c).

### 2.4. Immunohistomorphometry Does Not Reveal Changes in mMCP6 Expression

Immunofluorescence histomorphometry revealed no significant mMCP6+ MC difference between all treatment groups (Figure 2d). Differences in staining pattern and intensity as seen for mMCP4 could be detected over all groups, but without significance (Figure 2e,f).

### 2.5. Skipping the Immune Balance and Positive Correlation of mMCP4+ MCs and Indicators of Disease Severity

**Figure 3:**
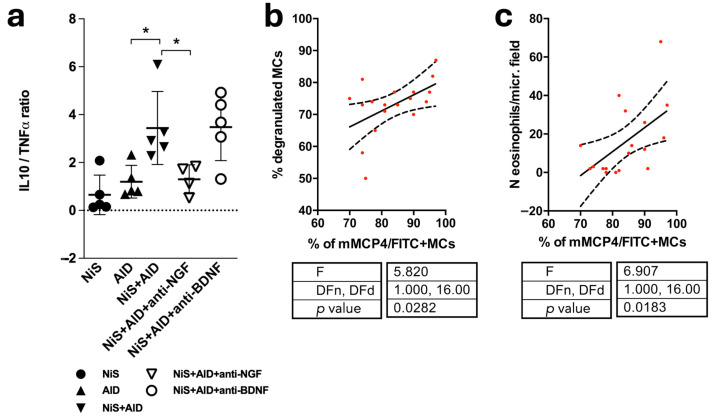
Pro-inflammatory cytokine imbalance and mMCP4+ MC percentage interacts with indicators of MC activation and allergic inflammation. Results are given in the figure. (**a**) IL10/TNFα mRNA expression ratio: mRNA levels were analyzed in full-thickness skin biopsies from experimental mice. Significant group differences were calculated by the Kruskal–Wallis Test. (**b**,**c**) Linear regressions were calculated, including 3–4 samples from each of the 6 experimental groups. Best-fit line and 95% confidence band are shown. * = *p* ≤ 0.05. (**b**) MC degranulation was used as a marker for stress-sensitive neurogenic inflammation. (**c**) Eosinophilic infiltration was used as a marker for allergic inflammation. Abbr.: AlD = atopic dermatitis-like allergic inflammation; anti-BDNF = antibody against BDNF; anti-NGF = antibody against NGF; BDNF = brain-derived neurotrophic factor; IL10 = interleukin 10; MC = mast cell; NGF = nerve growth factor; NiS = noise induced stress; TNFα = tumor necrosis factor alpha.

Finally, stress has previously been shown to skew the cutaneous immune balance and increase mast cell degranulation, thereby worsening cutaneous inflammation as evidenced by eosinophilic infiltration in the herein employed mouse model [15,16,33,35]. For the calculation of the balance between pro- and anti-inflammatory cytokines in murine back skin, we determined TNFα and interleukin 10 (IL10) mRNA levels in full-thickness skin biopsies and found a pronounced pro-inflammatory imbalance in NiS+AlD skin that was not detectable in mice additionally treated with aNGF (Figure 3a). To learn if mMCP4 expression associates with disease activity, the percentage of mMCP4+ MCs was correlated with the percentage of degranulated MCs and with the number of eosinophilic granulocytes, respectively. This revealed a positive correlation with both measures of disease severity (Figure 3b,c).

### 2.6. In Cultured MC, SP Induces MCP4 mRNA in the Presence of Chrna7

**Figure 4:**
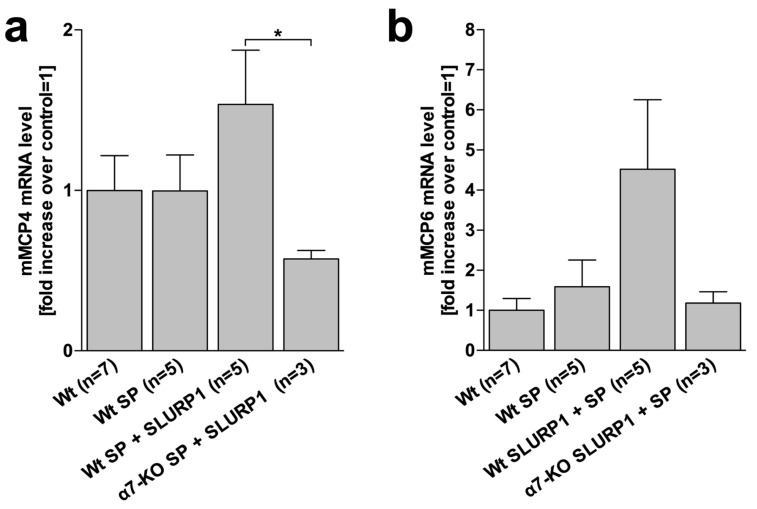
SP induces mMCP4 mRNA in the presence of Chrna7 in cultured MCs. (**a**,**b**) qRTPCR analysis of mMCP4 and mMCP6 mRNA expression in primary cultures of peritoneal MCs from Wt and α7-KO mice. Individual treatments are mentioned at the X axis of the graphs. Gene expressions were normalized to the housekeeping gene TBP. Data represent the averages of at least 3 different experiments. * = *p* ≤ 0.05. Abbr.: α7-KO = Chrna7 knockout; mMCP = murine mast cell protease; SLURP1 = Ly-6/uPAR-related protein 1; SP = substance P; Wt = wild type.

Peritoneal MC culture was employed to investigate if the observed in vivo changes of MC mMCP expression in response to stress could be triggered by SP and if Chrna7 and/or SLURP1 interfere with stress-mediator-induced MC mMCP expression. Analysis of mMCPs by qRTPCR revealed that SP alone did not alter mMCP4 mRNA production in cultured MCs compared to the wild type (Wt). By contrast, treatment of MCs with SP in the presence of SLURP1 resulted in high levels of mMCP4 mRNA production, which were absent in MCs from Chrna7 knockout (α7-KO) mice (Figure 4a).

### 2.7. No Significant Effect of SP on MCP6 in Cultured MC

Analysis of mMCP6 mRNA production revealed no significant changes between treatment groups, albeit concomitant SP and SLURP1 treatment in Wt also generated the highest mMCP6 mRNA levels (Figure 4b).

## 3. Discussion

Taken together these results demonstrate that allergic inflammation upregulates mMCP4, whereas stress alone has no such effect. However, in the presence of AlD, stress further increases mMCP4. Neutralization of NGF, a neurotrophin that regulates sensory and cholinergic skin innervation and neuropeptide production, has previously been shown to neutralize the effect of stress in NiS+AlD mice [15,16]. Here, we show that it also reduces the stress-induced mMCP4 upregulation in allergic inflammation. The positive correlation of mMCP4 with markers of allergic inflammation activity confirms a dose effect of the interaction between inflammation and stress. The treatment of cultured MC with SP, a key stress mediator shown to induce MC activation both in healthy and AlD skin [15,29,36], demonstrates that the stress mediator alone does not upregulate mMCP4. Upregulation only occurs in the additional presence of Chrna7 or SLURP1, the cholinergic neuropeptide we previously identified to be responsible for hyperinflammatory responses to stress [29].

Previously, we were able to show that the transient and NGF-dependent upregulation of SP by stress exerts deleterious effects on AlD [15,16]. The expression of mMCP4 protein in skin MCs of NiS+AlD mice is complementary to this previously published pattern of SP immunoreactivity in the NiS+AlD model [15]. The shift from high to low mMCP4+ MC in NiS as well as in AlD mice compared to the control suggests increased release of this SP cleaving protease under conditions of increased neurogenic inflammation. When NiS and AlD come together, mMCP4 production is further increased, as evidenced by the increased percentage of high mMCP4+ MCs. These results suggest that MCs, while activated to promote innate immunity, simultaneously produce and release factors such as mMCP4, which initiate adaptive immune responses.

NiS+AlD mice are a useful model to study exposome-triggered and -exacerbated allergic inflammation, as the protocol allows exact timing of the stress hit as well as the allergen challenge hit in a two-hit paradigm [1,12,16,29,35]. Under conditions of allergic inflammation and stress, the SLURP/Chrna7-promoted upregulation of mMCP4 observed here seems to play an aggravating role in this TH2-driven inflammation model [34,37]. This further elucidates why TH2-driven allergic inflammation is promoted when the cholinergic system is called into action [38,39]. On the one hand, the upregulation of mMCP4 increases the capacity to restrict innate and TH1-driven inflammation by digesting, for example, the stress mediator SP. On the other hand and as observed in many other inflammation-triggering contexts, the herein presented results demonstrate that the resilience to innate and TH1-driven inflammation provided by the cholinergic axis can support inflammation in AlD and especially in AlD exposed to aggravating exposome factors. Translationally, these results are intriguing, as we were previously able to show that humans exposed experimentally to stress during the Trier social stress test (TSST) also show an upregulation of SLURPs [40]. There is hence a possible role for cholinergic mechanisms affecting MC activity in the well-known stress aggravation of atopic dermatitis in humans.

Taken together, MCs may play a dual role in the aggravation of allergic dermatitis: they can be triggered by stress to enhance innate immunity and TH1 responses, but by releasing the enzymes cleaving the triggering stress mediators, they also promote the establishment of a TH2 imbalance. Translational investigations will be necessary to clarify the functional relevance and therapeutic potential of the cholinergic axis in allergic inflammation [41]. Treatment strategies addressing the cholinergic axis may offer an intriguing new research and treatment field for allergic inflammation.

## 4. Materials and Methods

### 4.1. Animals

As described previously, female C57BL/6 mice, 7-8 weeks old (Charles River, Sulzfeld, Germany), were randomly sorted into six groups (5 animals per group) on arrival at the animal facility of Universitätsmedizin-Charité, Berlin, Germany [15,36]. Animal care and experimental procedures were approved by the institutional review board and conformed to the requirements of the state authority for animal research (LaGetSi, Berlin, Germany).

In addition, primary murine MCs were obtained from 24–40-week-old Wt and α7-KO mice with the permission of the state of Hesse, Administrative Council Giessen, according to section 8 of the German law for the protection of animals and conforming to the NIH guide for the care and use of laboratory animals. As Wt mice, the corresponding background strain (C57BL/6J) was used. Characterization and description of these KO animals can be found elsewhere [42]. All mice were housed under a 12/12 h light/dark cycle in temperature-regulated rooms (22–24 °C), with food and water provided ad libitum until experiments were performed.

### 4.2. Genotyping

All the animals used in this study were genotyped as described by Moser and colleagues [43]. Approximately 0.5 µg DNA was used for the Polymerase Chain Reaction (PCR) using a Kapa mouse genotyping Kit (Peqlab, Erlangen, Germany). The thermal cycler profile followed published protocols [33].

### 4.3. The NiS-AlD Model

To investigate the impact of stress on an allergen challenge resulting in Langerhans-cell and TH2-driven skin inflammation resampling atopic dermatitis in mice, we employed a stress model established in our laboratory and combined it with a model for intradermal ovalbumine challenge following a subcutaneous sensitization phase as described by others [44,45,46,47,48,49]. This model was chosen rather than the more frequently used epicutaneous sensitization and elicitation models, as it produces a robust allergic inflammatory response directly following the challenge. Since the stress exposure was to precede the allergic inflammation, the combination of these two hits could be well timed. As earlier described, mice within the AlD groups were sensitized to ovalbumin 20 µg (Grade VI, Sigma-Aldrich, Schnelldorf, Germany) on day 1 and 14 of the experiment via subcutaneous injection [15,45,50]. Induction of AlD was achieved by intradermal injection of 50 µg ovalbumin (Grade V, Sigma-Aldrich, Germany) on day 21. According to the stress protocol, mice were exposed to noise (300 Hz tone, emitted at irregular intervals four times per minute; rodent repellent device, Conrad Electronics, Hirschau, Germany) for a single 24 h period [15,17,36] (NiS). Stress exposure occurred directly before AlD induction. Control mice received everything but the allergen. Mice were killed directly after stress or 48 h after AlD provocation. This animal model was first described in 2008 [15]. Our previous publications employing this model demonstrated that 24 h noise stress effectively doubles cutaneous inflammation in allergen-challenged mouse skin involving neuropeptides such as SP and neurotrophins such as NGF [15,16].

Animals within the aNGF treatment group were injected, as described below, with 200 μL anti-NGF antibody (Sigma-Aldrich, Germany) at a dilution of 1:1500. The dosage of anti-NGF was based on previously published data [17]. Animals within the aBDNF treatment group were injected with 125 μL of anti-BDNF antibody (Promega, Mannheim, Germany) containing 50 μg/mL anti-BDNF as per the manufacturer’s datasheet. Dilutions were done with PBS. Injections were given twice intraperitoneally in stressed AlD mice, directly before and after stress exposure. The second injection took place 30 min before AlD provocation. In this study, the blocking of NGF was preferred instead of the blockade of NGF receptors, because NGF and its receptors, namely TrkA and p75NTR, act in a complex interplay that demands blockade of the ligand in an in vivo setting to determine appropriate interactions. Transgenic investigations could only focus on single receptors, founded on the lethality of the NGF knockout mice [51].

The animals in the control group were kept under the same conditions. Instead of ovalbumin provocation, the animals in the control group received NaCl 0.9% intradermally and NaCl 0.9% intraperitoneally instead of anti-NGF/anti-BDNF.

### 4.4. Tissue Collection

For immunohistochemistry (IHC), tissues were collected as described in our earlier publication [33]. Briefly, for skin biopsies, PCR mice were deeply anesthetized and killed via cervical dislocation. Skin on the back was dissected under sterile conditions and quickly shock-frozen in liquid nitrogen. For skin biopsies intended for IHC, the fixative (LANA solution, composed of paraformaldehyde, NaOH, saturated picric acid, and distilled water) was injected using a butterfly syringe inserted through the left ventricle of the heart, and skin was harvested [16,52].

### 4.5. Immunohistochemistry

IHC was performed with a 14 μm thick mouse back skin cryostat section as described elsewhere [33]. The primary antibodies used in this study are c-Kit (1:100, Acris Antibodies, Herford, Germany), mMCP4 (1:400, Biozol, Eching, Germany), and mMCP6 (1:800, AF3736, R&D Systems, Minneapolis, MN, USA). FITC-labeled Avidin (Dianova, Hamburg, Germany) was used as the specific cutaneous MC marker [53]. Detailed information on the calculations is given in the corresponding figure legend.

### 4.6. Determination of Disease Severity

To determine the severity of inflammatory responses in NiS and AlD, the number of eosinophilic granulocytes and degranulated MCs were counted, and the percentage of degranulated MCs of the total MC number was calculated per the microscopic field and mouse, as described previously [15,35].

### 4.7. Peritoneal Mast Cell Culture (PMCC)

On day 0 of the experiment, mice were deeply anesthetized and killed with cervical dislocation. Abdominal skin was washed with 70% ethanol. An incision was then made in the ventral skin without disrupting the peritoneum, followed by an injection of 5 mL of air and NaCl 0.9% with a sterile syringe in the peritoneal cavity. Mice were shaken for at least 3 min for washing the peritoneal cavity, and the lavage was obtained with a fresh syringe and transferred to a 15 mL centrifuge tube. The peritoneal lavage was then centrifuged for 10 min at 4 °C and 1200 rpm. The supernatant was discarded, and the MC pellet was resuspended with PMCC medium (RPMI medium containing 5% fetal calf serum, 1% penicillin-streptomycin, 0.001% α-Monothioglycerol, 10 ng/mL Interleukin-3, and 30 ng/mL Stem cell factor). The centrifugation step was repeated one more time. MCs in RPMI were then transferred to a cell culture flask and stored in an incubator (37 °C and 5% CO_2_). The medium was changed on days 2, 5, and 8. On day 10, MCs were counted using a Neubauer chamber and the trypan blue exclusion principle. Around 300,000 cells were seeded in each well of a 6-well plate. Cells were treated with SLURP1 or SP and SP+SLURP1 for 24 h (see Table 1 for detailed description). Cells were also utilized for MC identification by cytospin followed by Giemsa and IHC of the MC marker c-Kit. After the treatment period, the cell pellets obtained from centrifugation were frozen at −80 °C for further total RNA isolation. Proliferation and apoptosis of PMCC were measured using mRNA expression of proliferation cell nuclear antigen and Caspase 3. The expression of c-Kit mRNA was also measured to confirm MC identity.

### 4.8. RNA Isolation and qRTPCR

For RNA isolation from PMCC, cell lysates were directly dissolved in RLT buffer and continued as described earlier [33]. The specific forward and reverse primers and Taqman probes (TIB Molbiol, Berlin, Germany) used in this study are described in Table 2. Gene expressions were normalized to the internal control TATA box binding protein using a modified version of the ∆∆CT method [54]. The means of control animal samples were set to 1.

### 4.9. Statistical Analysis

Statistical differences between the averages of the treatment groups were determined by one-way ANOVA with a post hoc Mann–Whitney U test. The association of indicators of neurogenically enhanced allergic inflammation with the MCP4 expression of mast cells was analyzed by linear regression analysis. All analyses were done with GraphPad Prism V.6 software (https://www.graphpad.com; accessed on 14 October 2014), and *p* < 0.05 was interpreted as significant.

## 5. Conclusions

To our knowledge, this is the first report about the counterregulatory upregulation of stress mediator cleaving MCPs in stressed skin. Further studies are necessary to investigate their pharmacological potential in controlling the skins stress response.

## Figures and Tables

**Table 1:** Substances used in PMCC.

Substance/Company	Concentration	Action
Substance P (Sigma-Aldrich, St. Louis, MA, USA, Cat No. S6883)	1 µM	Stressor
SLURP1 (Abnova, Taipei, Taiwan, Cat No. H00057152-P01–10 µg)	5 ng/mL	Chrna7 agonist

**Table 2:** Primers and probes used for qRTPCR.

Gene	Sequence
Caspase 3	Forward: GCATTGAGACAGACAGTGGGAC Reverse: CTCCAGGAATAGTAACCAGGTGC Taqman: 6FAM-TAAGCATACAGGAAGTCAGCCTCCACCG--BBQ
c-Kit	Forward: CCTTTCTGGTGTCCAACTCTGAT Reverse: AGATACATTCTGGACCTGTACGTCC Taqman: 6FAM-CCAGTGCTTCCGTGACATTCAACGT--BBQ
IL10	Forward: GACTTTCTTTCAAACAAAGGACC Reverse: GCTTGGCAACCCAAGTAA Taqman: 6FAM-ACTGCTAACCGACTCCTTAATGCAGG- -BBQ
mMCP4	Forward: GGCTGGAGCTGAGGAGATTATT Reverse: AGTGTGCAGCAGTCAACACAAAT Taqman: FAM-CCACTGAGAGAGGGTTCACAGCTACCTGT-BBQ
mMCP6	Forward: GTGCAGCTTCGTGAGCAGT Reverse: GGTGGGAGAGGCTCGTCATTAT Taqman: FAM-AGCTCCTCTCTTTGAACAGGATCGTGGT-BBQ
PCNA	Forward: CAACTTGGAATCCCAGAACAGG Reverse: GAACAGGCTCATTCATCTCTATGGTTA Taqman: 6FAM-TTGCACGTATATGCCGAGACCTTAGCCA--BBQ
TBP	Forward: GTGAATCTTGGCTGTAAACTTGACCT Reverse: GCAGTTGTCCGTGGCTC Taqman: 6FAM-AAATGCTGAATATAATCCCAAGCGATTTGC--BBQ
TNFα	Forward: GCCTATGTCTCAGCCTCTTCTCATT Reverse: CCACTTGGTGGTTTGCTACGA Taqman: 6FAM-CCATAGAACTGATGAGAGGGAGGCCATTT- -BBQ

## Data Availability

Data are contained within the article.
